# Wilson's Disease Manifestation in Late Adulthood: A Case Report and Literature Review

**DOI:** 10.7759/cureus.29797

**Published:** 2022-09-30

**Authors:** Faria Tazin, Harendra Kumar, Vern Orlang

**Affiliations:** 1 Family Medicine, East Liverpool City Hospital, East Liverpool, USA; 2 Medicine, Dow University of Health Sciences, Karachi, PAK

**Keywords:** atp7b mutation, wilson`s disease, internal medicine, genetics, family medicine

## Abstract

Wilson's disease is a rare inherited condition that results in an excessive copper buildup in various organs, especially the liver, brain, and other vital organs, leading to cirrhosis, liver failure, neurological problems like involuntary movements, clumsy gait, speech difficulties, and psychological issues, in addition to other symptoms. It is an ATP7B gene mutation-driven autosomal recessive condition. Although the condition is present from birth, symptoms often start to show up between the ages of five and 35, when the body has accumulated enough copper. Wilson's disease may become fatal if an excessive amount of copper is accumulated in the body. It is treatable and has a good prognosis if diagnosed early. Early identification, however, is not always straightforward since symptoms might resemble those of other diseases and develop later in life when copper is absorbed by food and drink and gradually accumulates in the body's many organs. In addition to medicine, physicians advise people with Wilson's disease to avoid foods and beverages containing copper. Our patient was diagnosed with Wilson’s disease at the age of 16 and was on Cuprimine which helped her to survive. Her younger sister was diagnosed at an early age and was started on a treatment regimen, which is why her sister’s manifestation of Wilson’s disease was less severe than hers.

## Introduction

Wilson's disease (WD), an autosomal recessive disorder that impairs copper metabolism, affects the ATP7B gene [1]. Copper tends to build up in the liver, brain, kidneys, and cornea in WD, leading to a variety of symptoms including hepatic illness, neuronal degeneration in the brain, and Kayser-Fleischer (KF) rings at the corneal limbus [2]. An indication of WD includes low copper levels in the blood and ceruloplasmin, increased excretion of copper in the urine, and increasing hepatic copper levels [3]. Contrarily, successful molecular testing is still diagnostic [1]. Low levels of copper in the blood and ceruloplasmin, as well as elevated levels of copper in the liver and increased excretion of copper in the urine, are characteristics/markers of WD [4]. In dire circumstances, liver transplantation could be a possibility [1]. We are reporting a case of a 59-year-old female with Wilson’s disease presenting in her late adult life.

## Case presentation

A 59-year-old female, with a past medical history of Wilson's disease (diagnosed at the age of 16), came to the clinic for evaluation of pain all over her body. As per the patient, the pain was noted to be significant over small joints of the hand, elbow, and knee joint. She described the pain as constant in nature with no aggravating or relieving factors and rated it 5/10 on the pain scale. She had a history of total hip arthroplasty, right knee arthroplasty, and left shoulder arthroplasty. She also presented with choreiform movements of her hands and legs, speech difficulty, and anxiety. Patient notes her younger sister was also diagnosed with Wilson's disease at an early age after her diagnosis as a screening process for genetic disease. On physical examination, patient appeared anxious, exhausted, and in mild distress. She is noted to be edentulous. The patient used a cane for walking and wore a brace on her right knee. On examination, patient's gait was slowed, clumsy, and choreiform movement was present. There was a deformity in the hands, fingers, elbows, and knee joints. Heart and lung sounds were normal. The abdomen was soft, non-distended, and non-tender in all four quadrants.

On neurologic examination, the patient had speech difficulties, and her speech was not comprehensible by the examiner. Her vitals were within normal limits. Her last lab work, including complete blood count (CBC), comprehensive metabolic panel (CMP), thyroid stimulating hormone (TSH), vitamin B12, and folic acid, was within normal limits. In Table 1, the hepatitis panel (HEP) of the patient shows high alkaline phosphate and low free T4. Whereas in Table 2, vitamin B6 and vitamin B12 are high. Moreover, ceruloplasmin and vitamin D were low. 

**Table 1:** The hepatitis panel (HEP) of the patient showed high alkaline phosphatase and low free T4

Test	Result	Flag	Reference
Uric acid	3.6		2.6 - 6.0 mg/dl
Phosphorous	3.9		2.5 - 4.9 mg/dl
Magnesium	1.9		1.5 - 2.1 mg/dl
Total protein	7.9		6.4 - 8.2 mg/dl
Albumin	3.3		3.1 - 4.5 mg/dl
Total bilirubin	0.4		0.2 - 1.0 mg/dl
Direct bilirubin	0.2		0.0 - 0.2 mg/dl
Serum glutamic-oxaloacetic transaminase (SGOT) / Aspartate aminotransferase (AST)	27		3 - 35 IU/L
Serum glutamic pyruvic transaminase (SGPT) / Alanine transaminase (ALT)	22		12 - 78 U/L
Alkaline phosphatase	132	High	45 - 117 U/L
Thyroid-stimulating hormone (TSH)	3.28		0.358 - 4.75 uIU/ml
Free thyroxine (T4)	0.75	Low	0.76 - 1.46 ng/dl

**Table 2:** Different tests showing levels of ammonia, vitamin B6, ceruloplasmin, vitamin B12, folic acid, and vitamin D in the patient

Test	Result	Flag	Reference
Ammonia	16		11-32 umol/l
Vitamin B6	53.8	High	2.0 – 32.8 ug/l
Ceruloplasmin	< 3.0	Low	19.0 – 39.0 mg/dl
Vitamin B12	1267	High	247-911 pg/ml
Folic acid	16.05		>5.38 ng/ml
Vitamin D	26.4	Low	30 – 100 ng/ml

Due to her movement difficulties, she had multiple falls in the past, which prompted several visits to the emergency room. A fall that caused a deep-cut wound in the elbow complicated her last Emergency Room (ER) visit due to sepsis. She was diagnosed with osteoarthritis, chronic pain syndrome, generalized anxiety disorder, and Wilson's disease. She was treated with Cuprimine (penicillamine) and Percocet (a combination of oxycodone and acetaminophen) with regular outpatient follow-up visits. 

## Discussion

An autosomal recessive condition known as hepatolenticular degeneration or Wilson's disease (WD) disrupts copper metabolism and manifests as a range of clinical symptoms. Copper is required in trace amounts for a variety of vital tasks, including neurotransmitter production, absorption of iron, free radical detoxification, and connective tissue development in cells [5]. More copper is absorbed than is required that the liver stores until needed and of which, extra amounts are expelled via the biliary system to maintain a healthy balance [6]. If ignored, Wilson's disease prevents the liver from eliminating extra copper, leading to organ malfunction and liver failure [6]. As the condition progresses, copper builds up in other bodily organs, such as the brain, eyes, kidneys, and heart, harming the organs in question [1,7,8].

The liver's working cells, known as hepatocytes, are in charge of creating ceruloplasmin, an enzyme containing six copper atoms that circulate the bulk of plasma copper throughout the body. Additionally, extra copper is transported by hepatocytes to the biliary system before being eliminated in the stool. A mutation in the ATP7B gene, which inhibits copper from binding to ceruloplasmin and prevents extra copper from being carried into the bile, results in Wilson's disease [1]. As a result, a potentially harmful quantity of copper gradually builds up in the liver, resulting in liver disease, hepatocellular damage, and eventually, copper transit into the bloodstream, which leads to a toxic copper buildup in other organs [1]. Hepatocellular injury may lead to the development of cirrhosis, hepatomegaly, acute liver failure, hepatitis, and chronic active hepatitis [1,5]. When copper builds up in the brain, it often affects the brain stem, basal ganglia, and cerebellum. Wilson's disease typically affects the brain after the liver, therefore many patients do not first exhibit liver dysfunction. Instead, the initial clinical signs are neurological and mental disorders [9].

The most common neurological symptoms noted in WD are "tremors, incoordination, dystonia, stiffness, difficulties with fine motor movements, and dysarthria," but they vary in severity from moderate to severe [6]. Depression, rage outbursts, delusions, sexual exhibitionism, hyperactive conduct, and paranoia are a few other common mental disorders noted in patients with WD [6]. Furthermore, several studies have shown "atrophy of the brain and cerebral white matter" [9].

Wilson's disease is challenging to diagnose due to the wide range of nonspecific clinical symptoms reported by patients and the broad range of age of those affected. It is conceivable that someone could remain asymptomatic, and the illness would not become apparent until much later in life. Because it takes time for sufficient copper to accumulate and reach levels high enough to interfere with organ function, this illness's symptoms often appear in adolescence and the early stages of adulthood. However, this sickness has lately been seen in people as young as three and as old as 72 [1,5]. The quantity of copper built up in the body directly relates to the symptoms' severity. The presence of undetectable serum ceruloplasmin and a hepatic copper value of 356 µg/g dry weight can be used to establish the diagnosis of WD [1]. Wilson's disease is challenging to diagnose, so it may take up to two years before treatment starts after the onset of clinical symptoms [1]. The patient may become helpless and immobilized in severe instances of this brain condition. Furthermore, unless the patient has a liver transplant, extreme examples of this liver condition may be deadly. Therefore, therapy must be started after a diagnosis as soon as possible since early identification and treatment may reverse symptoms and avert potentially fatal liver and brain damage.

WD is identified by various clinical signs, such as liver disease, neurological or mental issues, the appearance of Kayser-Fleischer rings, low serum ceruloplasmin levels, and, in more severe instances, significant urine copper excretion. The main imaging modalities for this condition include sonography, magnetic resonance imaging, and computed tomography. Wilson's disease is seldom diagnosed just based on the liver's sonographic appearance since these indications of liver illness are generic; further diagnostic tests such as liver biopsy are often carried out. Splenomegaly is often recognized as well [1,7,10,11]. However, there have been cases of WD patients whose livers seemed normal after a sonogram [9]. The most regularly affected parts of the brain, notably the basal ganglia, are also evaluated using sonography and magnetic resonance imaging. Transcranial sonography indicates high echogenicity in basal ganglia-related regions [9,12,13]. Various clinical signs and symptoms accompany Wilson's disease, but none are diagnostic in and of themselves; often, a correct diagnosis requires a combination of several presentations. The definitive diagnostic test is a liver biopsy that reveals high copper levels [1,6].

The patient must adhere to a lifetime regimen of medication, dietary changes, and other copper-prevention techniques after Wilson's disease is diagnosed. Chelation therapy is the first method to eliminate free copper in the circulation that isn't covalently attached to ceruloplasmin, commonly known as heavy metal detoxification [14]. A mixture of oral medications removes the extra copper, and the patient's blood copper levels are monitored often. Penicillamine was the first oral medication used in chelation therapy, but it produced a lot of unfavorable side effects, particularly among individuals with neurological symptoms. The second copper-chelating medicine to be developed, trientine, also had several serious adverse effects, including neurological issues. Wilson's disease is being treated with zinc. Zinc aids in lowering copper absorption in the intestines, as opposed to penicillamine and trientine, which work together to release copper from the body for urine excretion [6,14].

Zinc is an effective medication for preserving a healthy level of copper absorption and excretion after obtaining an average copper level. There are specific dietary restrictions, such as consuming no more than one milligram of copper daily, to be implemented. Avoiding whole wheat, organic meats, nuts, seafood, chocolate, and supplements high in copper would help maintain the levels of copper in the body. Additionally, strategies for reducing copper absorption in water must be devised. These include using only distilled water for drinking and cooking and installing copper removal devices in drinking water pipelines [6,14].

## Conclusions

Wilson's disease is an autosomal recessive and inherited disorder that accumulates copper throughout the body, especially in the liver, brain, and other vital organs. Due to the mutation of the ATP7B gene in Wilson's disease, copper transporting ATPase 2 protein cannot function properly, leading to excessive copper accumulation in different parts of the body. This causes various diseases, including liver failure, cirrhosis, and neurological, psychological, and kidney problems. Our patient was diagnosed with Wilson's disease at the age of 16 with various neurological and psychological issues. She was on Cuprimine and other treatment regimens, which helped her survive. Her younger sister was diagnosed with Wilson's disease at an earlier age than she was through genetic screening. That is why her sister had fewer clinical manifestations than her. It isn't easy to diagnose the case early as it takes time to accumulate copper in the body's vital organs, as copper is absorbed through foods and drinks. When the diagnosis of Wilson's disease is confirmed, we need to start medical treatment immediately to avoid life-threatening complications such as liver failure and cirrhosis, which may require a liver transplant for definitive treatment.

## References

[1] Mak CM, Lam CW (2008). Diagnosis of Wilson's disease: a comprehensive review. Crit Rev Clin Lab Sci.

[2] Liu G, Ma D, Cheng J (2018). Identification and characterization of a novel 43-bp deletion mutation of the ATP7B gene in a Chinese patient with Wilson's disease: a case report. BMC Med Genet.

[3] Huong NT, Lien NT, Ngoc ND (2018). Three novel mutations in the ATP7B gene of unrelated Vietnamese patients with Wilson disease. BMC Med Genet.

[4] Daneshjoo O, Garshasbi M (2018). Novel compound heterozygote mutations in the ATP7B gene in an Iranian family with Wilson disease: a case report. J Med Case Rep.

[5] Noble JA (2005). A case study: identifying a new case of Wilson's disease. J Am Acad Nurse Pract.

[6] Brewer GJ, Dick RD, Johnson VD, Brunberg JA, Kluin KJ, Fink JK (1998). Treatment of Wilson's disease with zinc: XV long-term follow-up studies. J Lab Clin Med.

[7] Sakaida I, Kawaguchi K, Kimura T, Tamura F, Okita K (2005). D-Penicillamine improved laparoscopic and histological findings of the liver in a patient with Wilson's disease: 3-year follow-up after diagnosis of Coombs-negative hemolytic anemia of Wilson's disease. J Gastroenterol.

[8] Hlubocká Z, Marecek Z, Linhart A, Kejková E, Pospísilová L, Martásek P, Aschermann M (2002). Cardiac involvement in Wilson disease. J Inherit Metab Dis.

[9] Ricciardi MC, Sirimarco G, Vicenzini E, Zuco C, Meco G, Di Piero V, Lenzi GL (2010). Transcranial sonographic findings in Wilson disease. J Ultrasound Med.

[10] Mortele KJ, Ros PR (2001). Imaging of diffuse liver disease. Semin Liver Dis.

[11] Akhan O, Akpinar E, Oto A, Köroglu M, Ozmen MN, Akata D, Bijan B (2002). Unusual imaging findings in Wilson's disease. Eur Radiol.

[12] Friedman LS, Gee MS, Misdraji J (2010). Case records of the Massachusetts General Hospital. Case 39-2010. A 19-year-old woman with nausea, jaundice, and pruritus. N Engl J Med.

[13] Walter U, Krolikowski K, Tarnacka B, Benecke R, Czlonkowska A, Dressler D (2005). Sonographic detection of basal ganglia lesions in asymptomatic and symptomatic Wilson disease. Neurology.

[14] Brewer GJ (2008). The risks of free copper in the body and the development of useful anticopper drugs. Curr Opin Clin Nutr Metab Care.

